# Simple and Cost-Effective Generation of 3D Cell Sheets and Spheroids Using Curvature-Controlled Paraffin Wax Substrates

**DOI:** 10.1186/s40580-024-00451-4

**Published:** 2024-10-31

**Authors:** Huijung Kim, Kyeong-Mo Koo, Chang-Dae Kim, Min Ji Byun, Chun Gwon Park, Hyungbin Son, Hyung-Ryong Kim, Tae-Hyung Kim

**Affiliations:** 1 Department of Biomedical Engineering, Institute for Cross-Disciplinary Studies (ICS), Sungkyunkwan University (SKKU), Suwon, 16419 Gyeonggi Republic of Korea; 2 Department of Intelligent Precision Healthcare Convergence, ICS, SKKU, Suwon, 16419 Gyeonggi Republic of Korea; 3 Center for Neuroscience Imaging Research (CNIR), Institute for Basic Science (IBS), Suwon, 16419 Republic of Korea; 4 School of Integrative Engineering, Chung-Ang University, 84 Heukseuk-Ro, Dongjak-Gu, Seoul, 06974 Republic of Korea; 5 Department of Pharmacology, College of Dentistry, Jeonbuk National University, Jeonju, 54896 Republic of Korea

**Keywords:** Paraffin wax, Cell sheet, Spheroids, Periodontal ligament cell, Mesenchymal stem cell, Quantum dots

## Abstract

The challenges associated with animal testing in pharmaceutical development have driven the search for alternative in vitro models that mimic human tissues more accurately. In this study, we present a simple and cost-effective method for generating 3D cell sheets and spheroids using curvature-controlled paraffin wax films, which are easily accessible laboratory materials that eliminate the need for extracellular matrix (ECM) components or thermo-responsive polymers. By adjusting the curvature of the paraffin wax film, we successfully generated human periodontal ligament fibroblast (HPdLF) cell sheets and bone marrow-derived mesenchymal stem cell (hBMSC) spheroids. Key parameters, such as cell density, substrate curvature, and incubation time, were identified as critical factors for optimizing the formation of these 3D structures. In addition, the use of quantum dots (QDs) for cell tracking enabled long-term visualization and distinction between different cell types within complex tissue-like structures. We further demonstrated that wrapping the hBMSC spheroids with HPdLF cell sheets partially replicated the connective tissue structure of the periodontal ligament surrounding the tooth root. This highlights the potential of this platform for the construction of more sophisticated tissue-mimicking assemblies. In conclusion, curvature-controlled paraffin wax films provide a versatile and practical approach for 3D cell cultures. This simplifies the generation of both cell sheets and spheroids, offering a promising tool for tissue engineering and regenerative medicine applications, where precise cell-to-cell interactions are essential.

**Graphical abstract:**

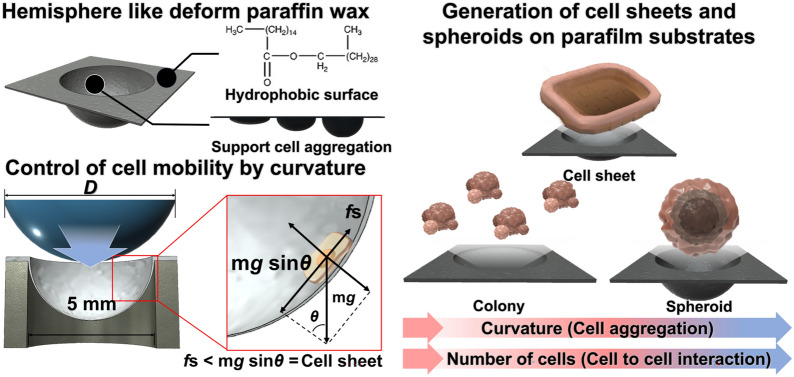

**Supplementary Information:**

The online version contains supplementary material available at 10.1186/s40580-024-00451-4.

## Introduction

Recently, animal testing in pharmaceutical development has faced challenges due to interspecies differences in cellular behavior, costly and labor-intensive maintenance, and ethical concerns [1–5]. To address these issues, mimicking tissue and organ structures in vitro has emerged as a promising alternative. Cellular composition, tissue microenvironments, and their functions have been the three major focuses when designing and generating in vitro organ-mimicking constructs [5–7]. To achieve multiple cell types, a variety of methods have been reported using multipotent stem cells as a core cell source, while various signaling molecules and proteins are employed to activate or inhibit cellular pathways during differentiation [8–10]. In the case of replicating the structural characteristics of target organs or tissues, it is essential to create three-dimensional (3D) assemblies that maintain the necessary cell-to-cell and cell-to-extracellular matrix (ECM) interactions [11–15]. The generation of such 3D cellular assemblies is also crucial for realizing tissue functions, which typically require the coordinated interplay of cells and ECM components [16–20]. However, conventional cell cultivation techniques rely on flat two-dimensional (2D) tissue culture plates (TCPs), which produce 2D-adhered cells with structures and cellular interactions that differ from those of tissues and organs. Therefore, converting adhered cells into 3D architectures, with the assistance of supporting materials, including biodegradable hydrogels, photo-crosslinkable polymers, or various ECM proteins, is essential.

Cell sheet is a thin mono- or multi-layered 2D cell assemblies wherein densely-packed cells are interconnected each other without the presence of underlying substrates [15, 21–23]. The main purpose of this cell sheet has been to treat damaged parts of tissues or organs, including heart [24], cornea [25], esophagus [26], periodontium [27], middle ear [28], knee cartilage [29], and lungs [30]. Recently, the cell sheet has shown potential as a basic building block for constructing scaffold-free in vitro models by stacking each layer with the help of biodegradable hydrogels or polymers [31–36]. To generate this cell sheet, one of the most frequently used method is the use of thermo-responsive polymer such as poly(N-isopropylacrylamide) (PIPAAm) [37, 38]. Specifically, at a typical cell cultivation temperature (37 ℃), PIPAAm is dehydrated (i.e., hydrophobic) and is thus highly adhesive to cell membrane, facilitating the formation of cell monolayers. However, when the temperature drops below 32 °C, the polymer becomes hydrated and swells, which reduces its adhesiveness and leads to the release of cell monolayers from the substrates. This reversible hydration and dehydration nature of PIPAAm in response to the temperature is highly advantageous for the generation of free-standing cell sheet without the need for cell detachment agents (e.g., trypsin–EDTA and proteolytic enzymes), which can lead to the loss or reduction of cell surface proteins [39]. Despite its effectiveness, several issues hinder the widespread adoption of thermo-responsive polymers for cell sheet engineering: (1) the complex experimental steps required to coat tissue culture plates (TCPs), including polymerization and immobilization of cell adhesion peptides [40]; (2) the high cost of the polymers and other reagents; and (3) the potential damage to adhered cells due to the temperature changes involved in cell sheet recovery [41–44]. Of these concerns, temperature-induced cellular damage is one of the most critical hurdles, as several RNA-binding proteins [45], including heterogeneous nuclear ribonucleoproteins (hnRNPs) [46] and cold-inducible RNA-binding proteins (CIRP) [47], as well as enzymes related to ATP production (e.g., ATPase and cytochrome c) [38], are known to be affected by low temperatures. These alterations in gene and protein dynamics may lead to reduced cell growth, viability, and function, all of which are crucial for accurately mimicking tissues and organs in vitro.

**Fig. 1:**
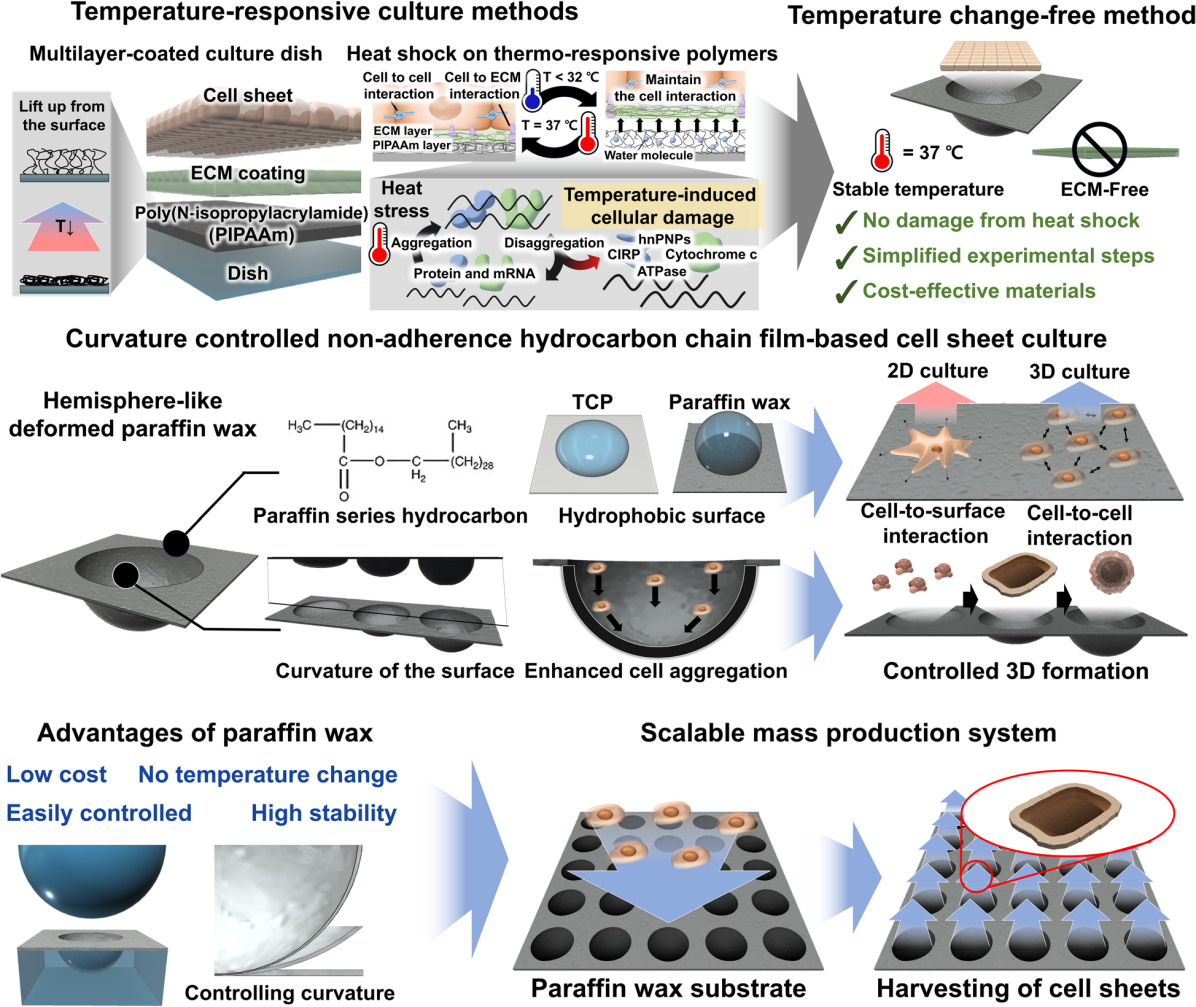
Schematic illustration of the study. Commonly used cell sheet culture methods are shown, which are based on thermosensitive polymers which modify proteins and genes and damage to cells that occur potentially due to heat shock. The surface properties, influence of paraffin wax, and adjuvant effect of cell aggregation on curvature are shown. Platform for the formation of selective three-dimensional structures, such as micro-sized cell sheets, with low cost and easy control

In this study, we present a simple, economical, and highly effective method for generating cell sheets without the need for temperature changes (Fig. 1). Parafilm, a widely used paraffin wax and polyolefin co-polymer commonly used for sealing laboratory glass and plastic wares, has recently been reported to inhibit cell adhesion, making it useful in generating organoids from stem cells [48–50]. Building on this property, we hypothesized that cell sheets could be generated using Parafilm when external force is applied to the cells. To test this, we deformed Parafilm using a metal spherical ball to optimize the curvature of its sidewall, thereby applying gravitational force to cells. We then varied the cell cultivation conditions, such as cell density and culture duration, on the deformed Parafilm, assessing the size and shape of the resulting cell sheets using human periodontal ligament fibroblasts (HPdLF) as a model cell line. Interestingly, we found that when the curvature was steeper than the optimized condition, cellular spheroids were formed instead of cell sheets. Based on these findings, we successfully generated both HPdLF cell sheets and human bone marrow-derived stem cell (hBMSC) spheroids using a simple curvature-controlled Parafilm, without the need for essential extracellular matrix (ECM) components or thermos-responsive polymers. Finally, the generated fibroblast cell sheets were treated with red-emitting quantum dots (QDs) and co-cultured with BMSCs to partially mimic the connective tissue structure that surrounds the tooth root within the bone socket.

## Experimental method

### Hemisphere-like deformation of the paraffin wax-based platform fabrication

The paraffin wax-based film was used as a commercialized product parafilm from Bemis (U.S.A.). The parafilm was cut to a width and length of 10 cm. This film and tip tray with 5 mm hole were washed with deionized (DI) water and 70% ethanol at 1 time. After washing, the parafilm and tray were sterilized under UV light for 30 min. Next, parafilm was placed on a tray and pressed down to mold a hole in the parafilm. Stainless-steel balls of different sizes (100, 50, 30, 10, 8, and 5 mm) were used for the bending of the pressing parafilm. The stainless-steel bare ball used at this time causes damage to paraffin when attached directly to the parafilm, minimizing damage to paraffin wax while minimizing changes in standards by using latex. Hemisphere-like deformation of the paraffin wax-based platform with 25-curvature was then transferred into a Petri dish containing 10 mL of Dulbecco’s phosphate-buffered saline (DPBS, Gibco). After, the petri dish containing the hemisphere-like deformation of the paraffin wax-based platform was transferred to a dish containing a larger size of DPBS to minimize the tilt of the platform that may occur further.

### Characterization of surface friction of paraffin wax

An experimental device for evaluating cell mobility was devised by referring to the tilting box method of A review on the angle of repose [51] and to fabricate a cell pathway to restrict the movement of cells in one direction in the following manner. The polyacrylate with a thickness of 130 µm is attached maintaining a gap between of 500 µm to both parafilm and a Petri dish. The polyacrylate with a gap between them was gently covered to prevent air droplets by the coverslip. After device was washed with deionized (DI) water and 70% ethanol at 1 time. After washing, the parafilm and tray were sterilized under UV light for 30 min. After sterilization the devices, HPdLF cells at a concentration of 50,000 cells/10 µL were injected into the empty space between the polyacrylate and incubation for 30 min under standard culture conditions (Fig. S1). Subsequently, the prepared samples were tilted incrementally from 0 cm and the movement of the cells according to the slope was measured using an optical microscope (Optinity KI-2000; Korea Lab Tech, South Korea). The interval of the inclination step was selected as 2 cm—the height at which cell movement was first observed. To provide stable support for the increasing slope, it was placed 1.5 cm inward from the bottom surface of the microscope. The final slope was 14.5 cm, which was the maximum height at which the center of gravity of the microscope could be maintained to avoid tipping over. Cell counts in the images were analyzed using ImageJ software (U.S. National Institutes of Health).

### Formation of 3D cell structures and cell culture

Before cell seeding, all reagents were of analytical grade. The solutions used in this study were prepared with DI water that were purified using a Millipore Milli-Q direct water purification system (EMD Millipore). All cell culture conditions were maintained at 37 °C, 5% CO_2_ in an incubator, and standard pressure and humidity conditions. HPdLF and hBMSC (Lonza Group Ltd., Basel, Switzerland) were cultured in 100 mm cell culture dishes. The medium was exchanged every 2 days after incubation. Subcultures were performed when 80% of the dish area was covered with cells. First, the medium was removed from the cells, after 2 mL of TrypLE (Gibco) was added and incubated at 37 °C for 3 min. The detached cells were collected and centrifuged at 1500 rpm for 3 min. After centrifugation, the cell pellet was resuspended by tapping and pipetting, after which 2 mL of DPBS was added for washing and centrifuged at 1500 rpm for 3 min. After repeating the washing process 2 times, the collected passages 4 to 5 of HPdLF and hBMSC were seeded at a calculated concentration of cell numbers per hole on the hemisphere-like deformation of the paraffin wax-based platform wax platform. The cells were cultured in Dulbecco’s modified eagle medium (DMEM, Gibco) with 10% fetal bovine serum (FBS, Gibco) and 1% antibiotic–antimycotic solution (Gibco).

The cell pellets from the washing process were resuspended in fresh medium according to the calculated concentration for each 3D cell culture method. After cell pellet was resuspended gently by tapping and pipetting taking care to avoid bubbles. For the hanging drop culture method, 50,000 cells in 20 μL of HPdLF and hBMSC growth medium (DMEM with 10% FBS and 1% antibiotic–antimycotic) were seeded on the cover of the cell culture dish. Inside the culture dish, 10 mL of DPBS was added to prevent evaporation from the cell droplets. The 3D spheroid culture was maintained under standard culture conditions (37 °C, 5% CO_2_) for 48 h. The spheroids were imaged at 0, 1, 3, 6, 12, 24, and 48 h using an optical microscope. For the hemisphere-like deformation of the paraffin wax-based platform used method, 100,000 cells in 20 μL of HPdLF and hBMSCs growth medium (DMEM with 10% FBS and 1% antibiotic–antimycotic, Gibco) on the hemisphere-like deformation of the paraffin wax-based platform transferred into a petri dish containing a 10 mL of DPBS. The manufactured spheroids and cell sheet 3D cell structures were used in the next experiment within 24 h.

### Quantum dot uptake optimization and fluorescence imaging analysis

HPdLF cells were cultured in a 6 well culture plate in growth medium, allowing them to proliferate to 80% confluency over a period of 2 days. The cells were washed 2 times with DPBS. After, incubate each well with a 2 nM concentration of Qdot™ 585 ITK™ Carboxyl Quantum Dots (Thermo Fisher, U.S.A.) in DMEM for 12 h. The size of the quantum dot used is ≤ 35 nm, and the Emission Maximum is found at 587 ± 4 nm. Cell viability analysis was evaluated using a CCK-8 assay (Dojindo, Kumamoto, Japan) and following the protocol of the manufacturer.

The cell sheet and spheroids were washed with PBS at 2 times. and fixed with 10% NBF solution overnight so that it can sufficiently fit into the interior of the 3D structure. After fixation, washed with PBS at 2 times. After each 3D cell structure samples were treated with 1% Triton X-100 diluted with PBS for 10 min. After, washed with PBS at 2 times. Washed samples were stained with Hoechst 33,342 (1 μg/mL, Sigma-Aldrich) for 10 min. The stained samples were transferred to a confocal dish and covered with cover-slide glass. Quantum dot (585 nm, red color) and nucleus-stained (460 nm, blue color) cells were performed using confocal fluorescence microscope (Leica, DMi8).

### Statistical analyses

All data were presented as mean ± standard deviation (S.D.) with three replication samples. The statistical significance between two groups was determined using the unpaired Student’s t test. Statistical significances in more than two groups were analyzed using one-way analysis of variance (ANOVA) with Tukey’s post hoc test. A significant difference is marked as * (p < 0.05), ** (p < 0.01), *** (p < 0.001), **** (p < 0.0001).

## Results and discussion

### Controlling cell movement and aggregation via spherically deformed paraffin wax film

**Fig. 2:**
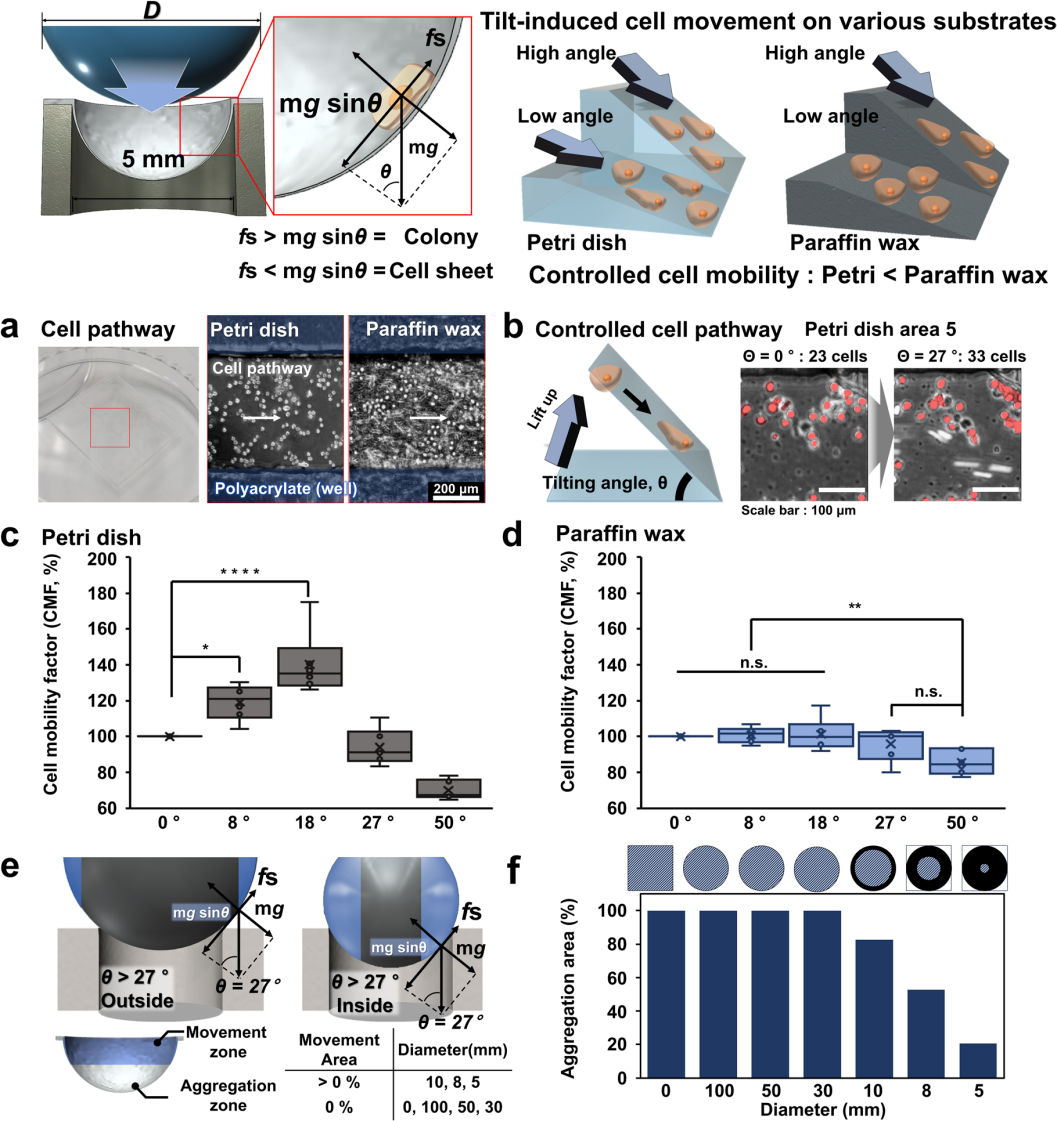
Stable curvature formation through paraffin wax. Image of curvature supporting to cell mobility and 3D structure formation and cell pathway for angle of repose (**a**). Cell counting according to controlled tilting angle of cell pathway. Only the cells that did not move in pathway were counted (**b**). Quantification of cell movement based on five different angles (0°, 8°, 18°, 27°, and 50°) controls on petri dish (**c**) and paraffin wax (**d**). Curvature-induced cell aggregation on an area of spheres to secure cell mobility and area that forms a stable angle according to curvature (**e**, **f**)

Paraffin wax is a long-chain hydrocarbons and exhibits low reactivity with chemical elements such as nitrogen and oxygen, rendering it highly stable and biocompatible with non-toxicity for cell culture applications [49, 52]. Possessing hydrophobic properties, it does not absorb water, making it suitable for cell culture environments [53, 54]. Specifically, it not only obstructs cell adhesion on hydrophobic surfaces but also introduces differences in cell mobility due to surface friction [55], which, in turn, affects the formation of 3D cellular structures based on cellular mobility [56]. Based on these interesting properties of paraffin wax, we hypothesized that applying curvature on the film will guide cells to move to specific direction, resulting in the cellular aggregation. First, to study the level of cellular mobility with respect to the curvature change, polyacrylate guides were fabricated on both typical TCPs and paraffin wax film for unidirectional cell movement along with cultivation medium. The angle was varied as 0, 8, 18, 27, 50 degrees with monitoring the changes of cell numbers at the fixed area where the optical microscope is focused on to capture the images (Figs. 2a, b, S1 and S2). The resulting cell mobility factor (CMF) was measured based on following equations (Fig. 2c and d).

$${\text{Cell Mobility Factor}} \left( \% \right) = \frac{{{\text{Number of cells remaining after tilting}} ({\text{Cr}})}}{{{\text{Initial number of cells (Ci)}} }} \times 100$$

In the case of TCPs, the model cell line, HPdLF, showed 19.18 ± 9.02 CMF at a relatively low angle of 8°. As the angle becomes steeper, CMFs were increased to 40.26 ± 16.26 and 6.01 ± 8.95, for 18° and 27°, respectively, indicating that cells are not sensitively affected by the TCP surface and move sensitively to the slight angular changes. Therefore, the result indicate that TCPs are not suitable for controlling cellular aggregation with curvature. In contrast, no statistical differences in CMFs were observed from the Paraffin wax film with the same experimental conditions: 0.91 ± 3.96 (8°) and 1.24 ± 8.29 (18°). The difference was observed when the angle reached 27° (CMF: 4.08 ± 8.32) and 50° (CMF: 14.47 ± 6.11). Based on this result, 27° is considered suitable for controlling cellular movement to specific direction with maintaining the anti-adherent property of paraffin was film. The force applied to the single cells is calculated to be 0.134 nN assuming the mass of the cells is 30 picograms as depicted in Fig. 2e [*F*_*g*_ = mgsin(θ), where m is a mass of single cell]. The viscous drag of the culture medium that is similar to the water is calculated based on following Stock’s law

$${\text{Viscous drag of the medium }}\left( {F_d } \right) = { 6}\pi \times \eta \times {\text{r}} \times {\text{v}}$$

where η, r, and v are the dynamic viscosity of the fluid (η = 0.001 N s/m^2^ for water), radius of the cells that is presumed to be spherical (r = 5 × 10^−6^ m), and velocity of the cells. The v is measured to be 0.9 × 10^−6^ m/s for this study, resulting *F*_*d*_ value of 0.085 pN, which is negligible compared to *F*_*g*_. Therefore, we can conclude that the cellular movement can be precisely controlled only based on the level of curvature. However, to generate cell sheet or spheroids, an initial cellular aggregation should be occurred that work as a seed for further cell assembly. To achieve this, we used metal spherical ball that can deform the paraffin as hemisphere-like structure. As shown in Fig. 2e, the deformed area can be roughly divided into two different regions based on the angle aforementioned: (1) the aggregation zone where cells are collected at the bottom of the well and are forced to generate small cellular aggregate and (2) the movement zone where cells fall to the aggregation zone, adhere to the existing cells, and keep generating cell sheet. The area where the cell movement within the deformed paraffin wax film was visualized and analyzed as shown in Fig. 2f. Areas with a slope of 27° or steeper that act as a critical angle for inducing cellular movement are marked with blue color. Owing to the thickness of the paraffin wax film, there was a limit to deform to maintain the mechanical stability of the film. In all curvatures using spheres with a size exceeding 10 mm, the angle generated at the sidewall of well was lower than 27°, making it difficult to induce cell movement and aggregation at the aggregation zone. Therefore, the spheres having diameter under 10 mm was finally chosen that provides the movement and aggregation zone with the ratio of 5:5.

### Simple 3D cell sheet generation using hemisphere-like paraffin wax film

**Fig. 3:**
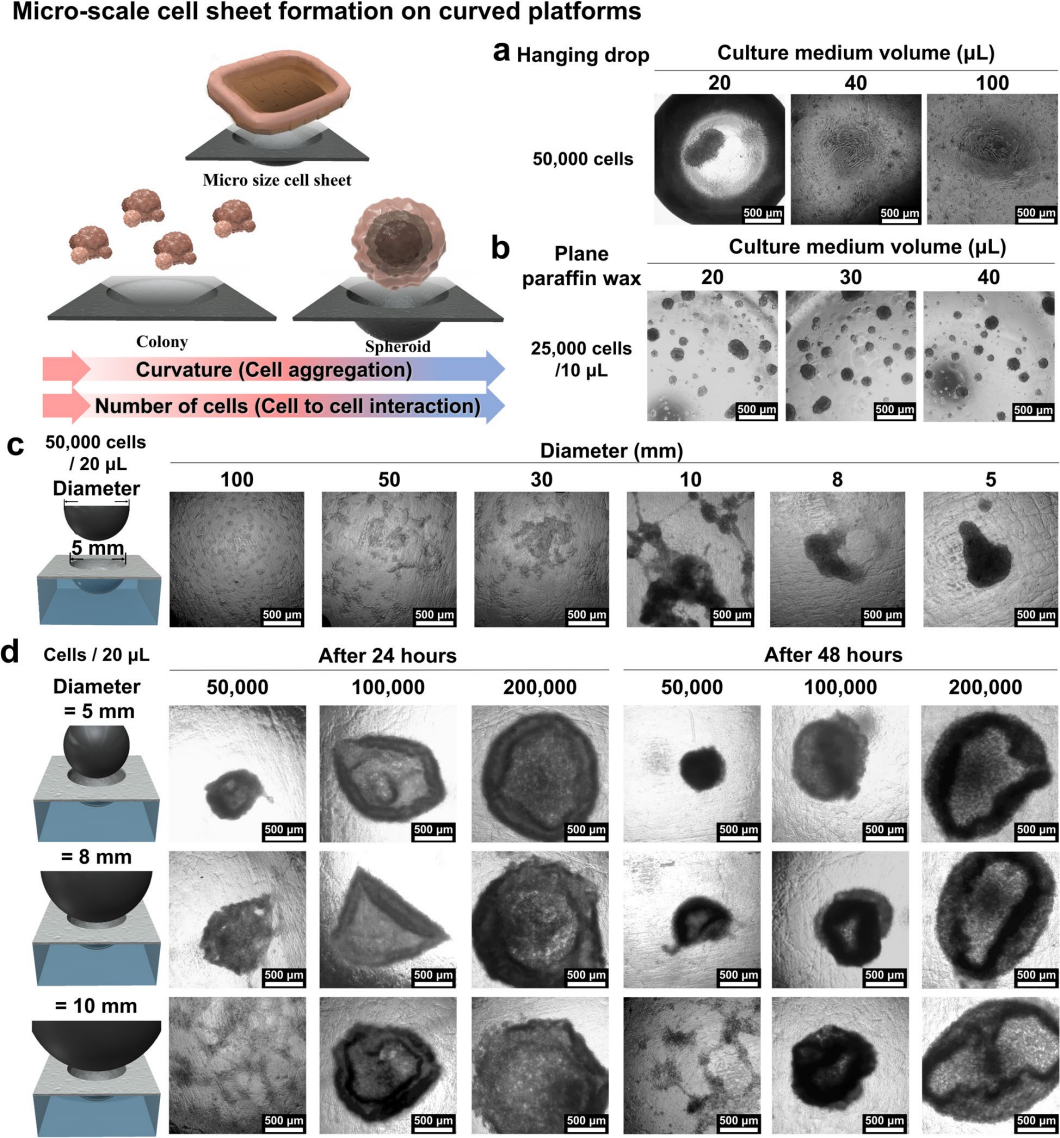
Optimization of 3D Cell Structure Formation. Cell-to-cell interaction modulated by culture conditions. Analysis of cell-to-cell interaction effect of cell aggregation by increasing the medium volume in the 50,000 cells for 24 h (**a**). 3D structure formation by increasing cell numbers (50,000 cells, 75,000 cells and 100,000 cells) with the same cell density (25,000 cells/25 μL) in a plane condition (**b**). Spheroid formation based on mold curvature (**c**). As the diameter of the sphere decreases from 100 to 5 mm, The aggregation of cells increases by according to curvature. Cell sheet formation as a function of curvature and cell density (**d**). To optimize the formation conditions of a cell sheet, three curvature conditions (5, 8, and 10 mm) and three cell concentrations (50,000, 75,000, and 100,000 cells/20 μL) conditions are combined and cultured for 2 days for imaging

To further explore the potential of the deformed paraffin wax film as a 3D cell cultivation platform, we compared its efficiency in generating cell sheets or spheroids with that of the conventional hanging drop method and a flat parafilm substrate. Approximately 20, 40, and 100 µL droplets of cell culture medium, each containing 50,000 cells, were formed and maintained for 24 h. As illustrated in Figs. 3a and S3, the hanging drop method at the 20 µL volume resulted in the formation of cell spheroids at the bottom of the droplet, likely due to the steep curvature of the droplet wall. However, at 40 and 100 µL volumes, neither spheroids nor cell sheets were observed, possibly due to the reduced cell density, which weakened cellular cohesion and cell-to-cell interactions. We also investigated the use of non-deformed flat paraffin wax film to evaluate its ability to support 3D cellular aggregation, leveraging its low cell adhesion properties. Under experimental conditions of 30, 40, and 50 µL of culture medium containing 25,000 cells, small, random spheroids or colonies were generated within a size range of 45–350 µm. This was due in part to the lack of external constraints that promote cellular cohesion and further aggregation (Figs. 3b and S4). When varying experimental conditions to include 50,000 cells/20 µL, 75,000 cells/30 µL, and 100,000 cells/40 µL, the resulting spheroid sizes were 99.28 ± 62.85 µm, 95.68 ± 42.33 µm, 108.56 ± 54.70 µm, and 99.28 ± 62.85 µm, respectively. These findings indicate that altering the medium volume while maintaining a fixed cellular density does not significantly influence spheroid size or shape.

Intriguingly, we identified the formation of a cell sheet-like structure at the base of the parafilm, which had been deformed by metal spheres with diameters ranging from 5 to 10 mm (Fig. 3c). This observation prompted us to systematically vary cell concentration and sphere diameter to investigate the potential for cell sheet generation without the need for ECM proteins or stimuli-responsive polymers. As demonstrated in Figs. 3d and S5–S7, a millimeter-scale cell sheet was effectively generated when the cell density reached approximately 100,000–200,000 cells. After 24 h of incubation, a 1.5 mm diameter cell sheet was formed, with 200,000 cells on the parafilm deformed by the 5, 8, and 10 mm metal spheres. While 100,000 cells were also able to form a smaller cell sheet, this structure began to curl at the edges after 48 h of incubation, eventually leading to spheroid formation. Conversely, the 200,000-cell sheet preserved its integrity over the same period, though slight edge curling was noted. These results suggest that initial cell concentration, hemisphere diameter of the parafilm, and incubation time are key parameters for optimizing cell sheet generation using the deformed paraffin wax film.

### 3D stem cell spheroid generation via controlling the curvature of paraffin wax

**Fig. 4:**
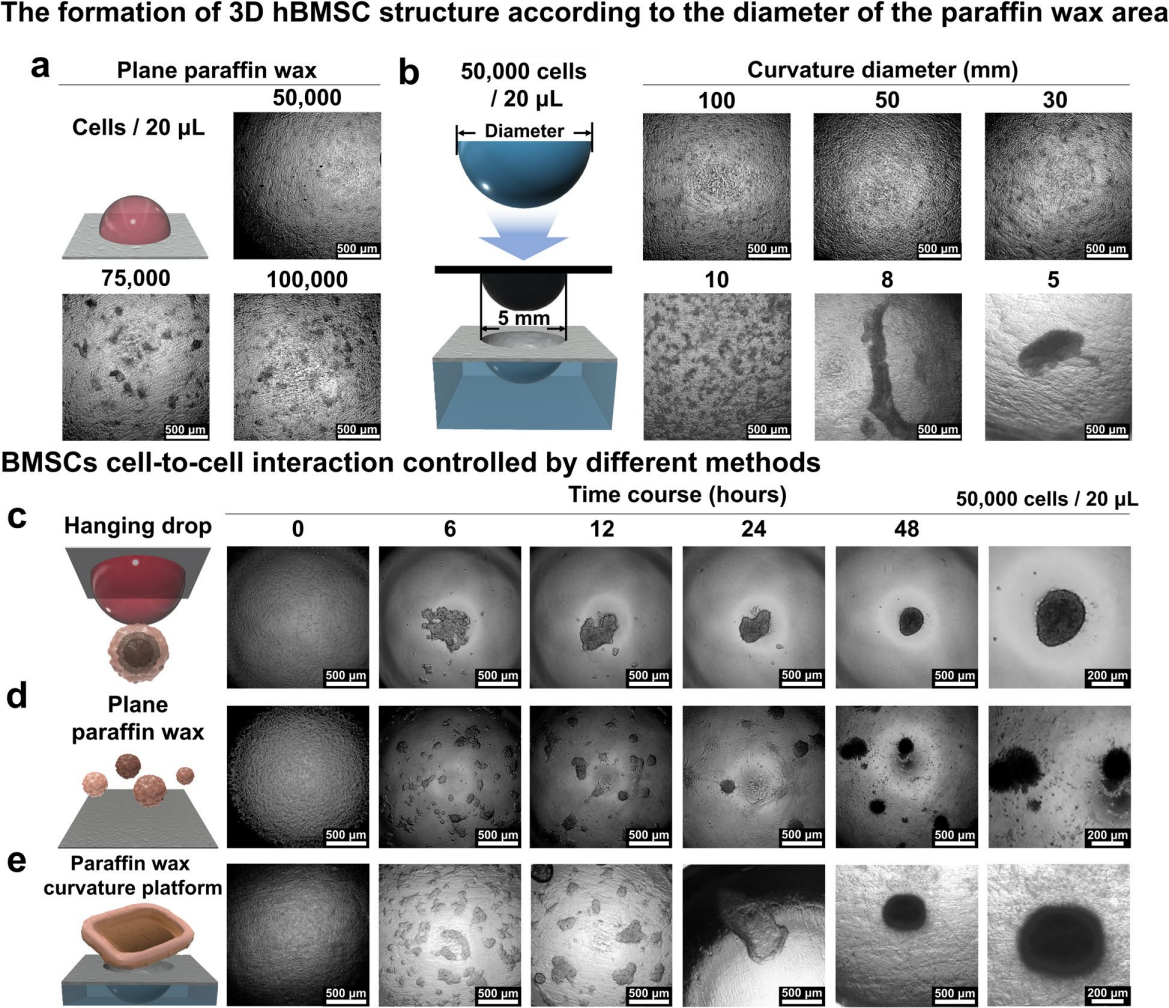
Optimization of 3D cell culture formation of hBMSCs on paraffin wax. formation of cell structures on a plane paraffin wax platform under conditions that the number of cells/20 μL is 50,000 cells, 75,000 cells, and 100,000 cells (**a**) and 50,000 cells/20 μL seeding condition on diameter of various curvatures from 100 to 5 mm (**b**) and commonly spheroid formation method and amounts of uncontrolled spheroid on plane paraffin wax flatform, and cell sheets formed on optimized platforms and aggregated spheroid formation over time (**c**–**e**)

Having established the potential of hemispherical paraffin wax for 3D cell sheet generation, our next objective was to investigate its application in spheroid generation using various cell types. For this purpose, we utilized human bone marrow-derived mesenchymal stem cells (hBMSCs), given their capacity to differentiate into odontoblast-like cells, which are crucial for mimicking tooth structure. This approach was explored alongside human periodontal ligament fibroblasts (HPdLF), which we previously used for cell sheet generation. Significant variability in the size and shape of spheroids was observed when they were generated solely through cell-to-cell interactions, without the influence of external forces or spatial constraints. To address this, we investigated spheroid generation on both planar and deformed paraffin wax films, focusing on their size and morphology. An initial seeding density of 25,000 cells/10 µL formed liquid droplets on the planar paraffin wax film, resulting in the formation of numerous random colonies, consistent with observations from HPdLF (Figs. 3b and 4a).

Next, we created hemisphere-like parafilm substrates using metallic spheres with diameters ranging from 5 to 100 mm. As shown in Fig. 4b, under short-term cultivation conditions (~ 24 h), the films deformed with 5 and 10 mm spheres facilitated cell aggregation, while diameters over 30 mm failed to produce any 3D cellular structures at a fixed cell density (50,000 cells/20 µL). This outcome is likely due to the weak cell-to-cell interactions, similar to those observed in the cell sheet study, governed by various cell membrane proteins such as E-cadherins, integrins, and selectins. However, due to differences in protein expression, hBMSCs exhibited less cellular aggregation than HPdLF under the same cell density and concentration.

Finally, we compared spheroid generation efficiency and variability in size and shape using three different methods: hanging drop, planar paraffin wax, and hemispherical paraffin wax (Figs. 4c-e and S8) with a fixed cell concentration (50,000 cells/20 µL). To induce strong cell-to-cell interactions crucial for spheroid formation, we selected a small sphere (5 mm in diameter) for film deformation. As illustrated in Fig. 4c and d, the conventional hanging drop method produced a single hBMSC spheroid with a diameter of 220 µm, while the planar paraffin substrate generated multiple irregular cell aggregates ranging from 50 to 200 µm. hBMSCs seeded on the hemispherical paraffin wax film exhibited sequential changes in cell aggregate morphology—from multiple small cell sheets to merged 3D cell aggregates, culminating in large spheroids with diameters of 350–400 µm (Fig. 4e). These observations led us to conclude that both cell sheets and cell spheroids can be effectively generated using HPdLF and hBMSCs by simply altering the curvature of the deformed parafilm.

### Wrapping hMSCs spheroids with HPdLF cell sheet to mimic the tooth root within the bone socket

**Fig. 5:**
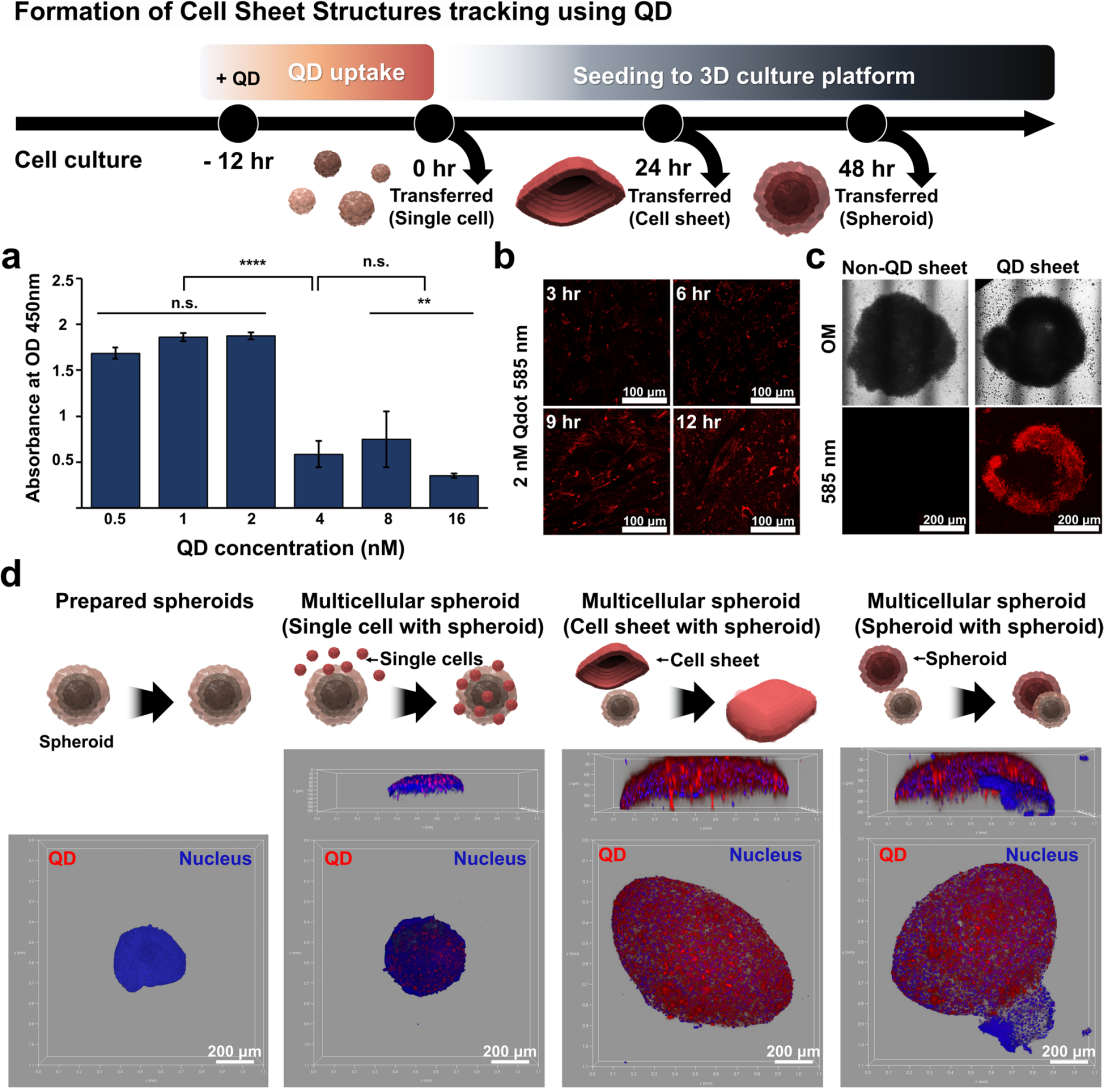
3D micro-sized cell sheet-like structures and multicellular spheroids tracking through quantum dots (QDs). Specific structure of cell sheets through QD fluorescence. Bar graph showing the cytotoxic effects of varying concentrations of Qdot-585 on HPdLF cells, with optical density (OD) measured at 450 nm. No significant cytotoxicity (n.s.) was observed at lower concentrations. and Significant cytotoxicity was observed above 4 nM (**a**). Fluorescence microscopy images of HPDLF cells over time (3, 6, 9, and 12 h) after treatment with 2 nM Qdot-585. The images depict the distribution and intensity of QD fluorescence within the cells (**b**). Comparison of non-QD treated cell sheets and QD-treated cell sheets under optical microscopy and fluorescence microscopy (585 nm). The QD-treated cell sheet shows distinct fluorescence, enabling clear visualization (**c**). Multicellular spheroid formation mixes pre-prepared spheroids with cell structures cultured in each form (single cell, cell sheet, spheroid). In the fluorescence image of the multicellular spheroids, the nucleus of all cells are stained blue fluorescence (460 nm), and only the QD-treated cells show red fluorescence (585 nm) (**d**)

After successfully forming HPdLF cell sheets and hMSC spheroids using paraffin wax films with varying curvatures, we advanced to the generation of a 3D multicellular tissue-like structure. Our goal was to wrap the spheroids with HPdLF cells to mimic the connective tissue structure of periodontal ligament tissue that surrounds the tooth root within the bone socket. However, due to the high cell density of the cell sheet and the 3D structure of the spheroids, distinguishing between these two cell lines after merging is challenging. Therefore, we utilized quantum dots (QDs) as a cell tracker because it can stably remain in the cytosol and maintain capability of emitting strong fluorescence under UV exposure for a long period of cultivation time [57, 58]. Carboxylated red-emitting quantum dots (QDs) with a diameter of approximately 5 nm were utilized, as the carboxyl groups on their surface are known to facilitate attachment to the cell membrane and promote uptake into the cytosol via clathrin-mediated endocytosis. Prior to treating the cell sheets with QDs, a cell viability test was conducted to determine the optimal concentration that would provide a strong fluorescence signal (i.e., high QD concentration) without inducing cytotoxicity. As demonstrated in Fig. 5a, the cytotoxic effects of QDs on cell sheets were confirmed at a concentration of 4 nM, which led to a decrease in cell viability by up to 74%. Consequently, 2 nM QDs were selected as the optimal concentration, providing strong fluorescence signals for up to a week without compromising viability (Fig. 5a and b). Remarkably, the QDs remained within the cell sheets even after multiple experimental procedures, including washing, fixation, and nuclear staining, as shown in Fig. S9 Following QD uptake, both individual cells and cell sheets were easily visualized over extended culture periods, a notable improvement over untreated cell sheet (Fig. 5c). Subsequently, we generated single cells, cell sheets, and spheroids on hemispherical parafilm substrates at days in vitro (DIV) 0, DIV 1, and DIV 2, respectively. As depicted in Fig. 5d, when QD-labeled HPdLF cells were seeded onto hBMSC spheroids, the HPdLF cells adhered to the spheroids as single cells, distinctly different from the formation of a tissue-like interfacial layer. Creating a connective tissue-like interfacial layer in vitro proved challenging in co-culture experiments, where incomplete fusion of HPdLF and hBMSC spheroids was observed. However, a structure resembling the periodontal ligament, where the tissue surrounds the tooth root, was partially achieved when hBMSC spheroids were enwrapped with an HPdLF cell sheet, clearly visible under fluorescence microscopy.

In conclusion, the curvature-controlled paraffin wax film not only simplifies the generation of both cell sheets and spheroids but also proves valuable in constructing connective tissue-mimicking structures, where close physical interaction between different cell lines is crucial.

## Conclusions

In this study, we developed a simple, cost-effective, and highly adaptable platform for generating 3D cell sheets and spheroids using curvature-controlled paraffin wax films. This approach eliminates the need for complex materials such as extracellular matrix (ECM) proteins or thermo-responsive polymers, making it an accessible alternative for various cell culture applications. By adjusting the curvature of the paraffin wax film and optimizing the cell cultivation conditions (e.g., cell density and number), we successfully generated HPdLF cell sheets and hBMSC spheroids, demonstrating that the physical properties of the substrate could be finely tuned to control cell aggregation and assembly. Finally, we demonstrated that this platform could be extended to more complex tissue-like structures by wrapping hBMSC spheroids with HPdLF cell sheets, partially mimicking the connective tissue structure of the periodontal ligament. Moreover, the use of quantum dots (QDs) as a cell-tracking tool proved effective in distinguishing different cell types within 3D constructs, offering a valuable method for the long-term visualization of cellular interactions. This capability is crucial for developing in vitro models that can replicate the structural and functional complexities of tissues and organs more accurately.

In conclusion, curvature-controlled paraffin wax films present a versatile and practical solution for 3D cell culture, offering significant potential for tissue engineering and regenerative medicine applications. This platform not only simplifies the generation of 3D cell sheets and spheroids but also holds promise for the construction of more sophisticated, tissue-mimicking structures where precise cell-to-cell interactions are essential.

## Supplementary Information

Additional file 1.

## Data Availability

Datasets used and/or analyzed during the current study are provided by the corresponding authors upon reasonable request.
