# Supplementary material for: Simple and Cost-Effective Generation of 3D Cell Sheets and Spheroids Using Curvature-Controlled Paraffin Wax Substrates
Source: Nano Converg. 2024 Oct 31;11:44. doi: 10.1186/s40580-024-00451-4 (PMC11527855; doi:10.1186/s40580-024-00451-4)
Supplement: Supplementary file 1 — Additional file 1. [file 40580_2024_451_MOESM1_ESM.docx]

Supplementary Information

**Simple and Cost-Effective Generation of 3D Cell Sheets and Spheroids Using Curvature-Controlled Paraffin Wax Substrates**

Huijung Kim^1,2,3^, Kyeong-Mo Koo^1,2^, Chang-Dae Kim^1,2^, Min Ji Byun^1,2^, Chun Gwon Park^1,2^, Hyungbin Son^4^, Hyung-Ryong Kim^5,*^ and Tae-Hyung Kim^1,2*^

^1^Department of Biomedical Engineering, SKKU Institute for Convergence, Sungkyunkwan University (SKKU), Suwon, Gyeonggi, 16419, Republic of Korea

^2^Department of Intelligent Precision Healthcare Convergence, SKKU Institute for Convergence, Sungkyunkwan University (SKKU), Suwon, Gyeonggi, 16419, Republic of Korea

^3^Center for Neuroscience Imaging Research (CNIR), Institute for Basic Science (IBS), Suwon 16419, Republic of Korea

^4^School of Integrative Engineering, Chung-Ang University, 84 Heukseuk-ro, Dongjak-gu, Seoul 06974, Republic of Korea

^5^Department of Pharmacology, College of Dentistry, Jeonbuk National University, Jeonju, 54896, Republic of Korea

***Corresponding author: Hyung-Ryong Kim (hrkimdp@gmail.com), Tae-Hyung Kim** **(thkim0512@****skku.edu)**


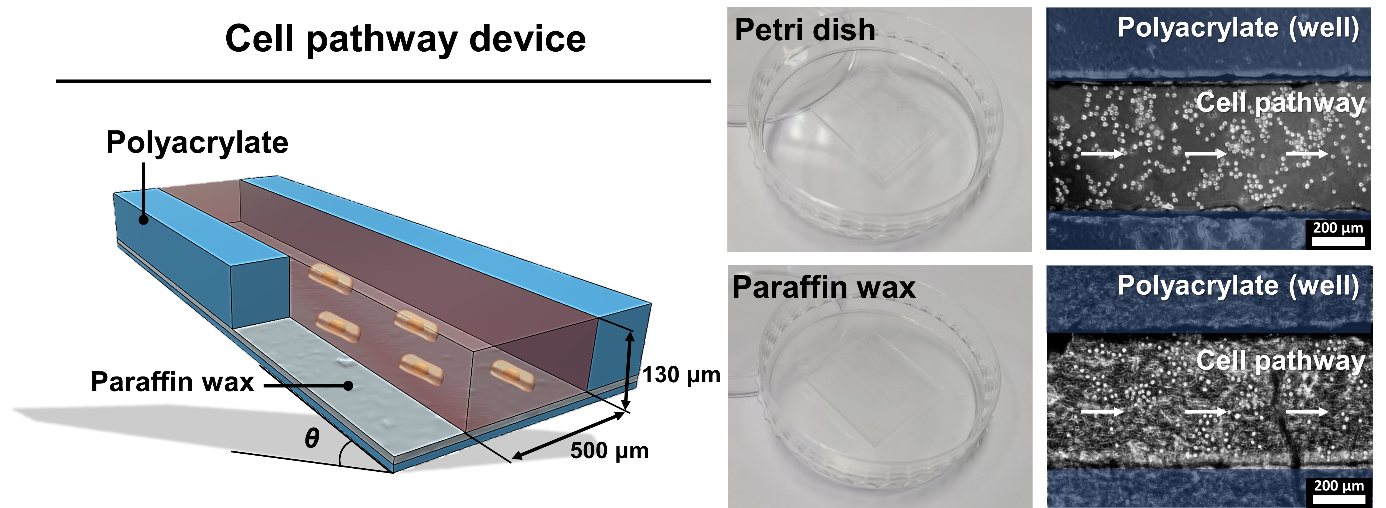


**Figure S1.** A schematic diagram illustrating the cell pathway using polyacrylate, along with optical images of cells along this pathway on a petri dish and paraffin wax.


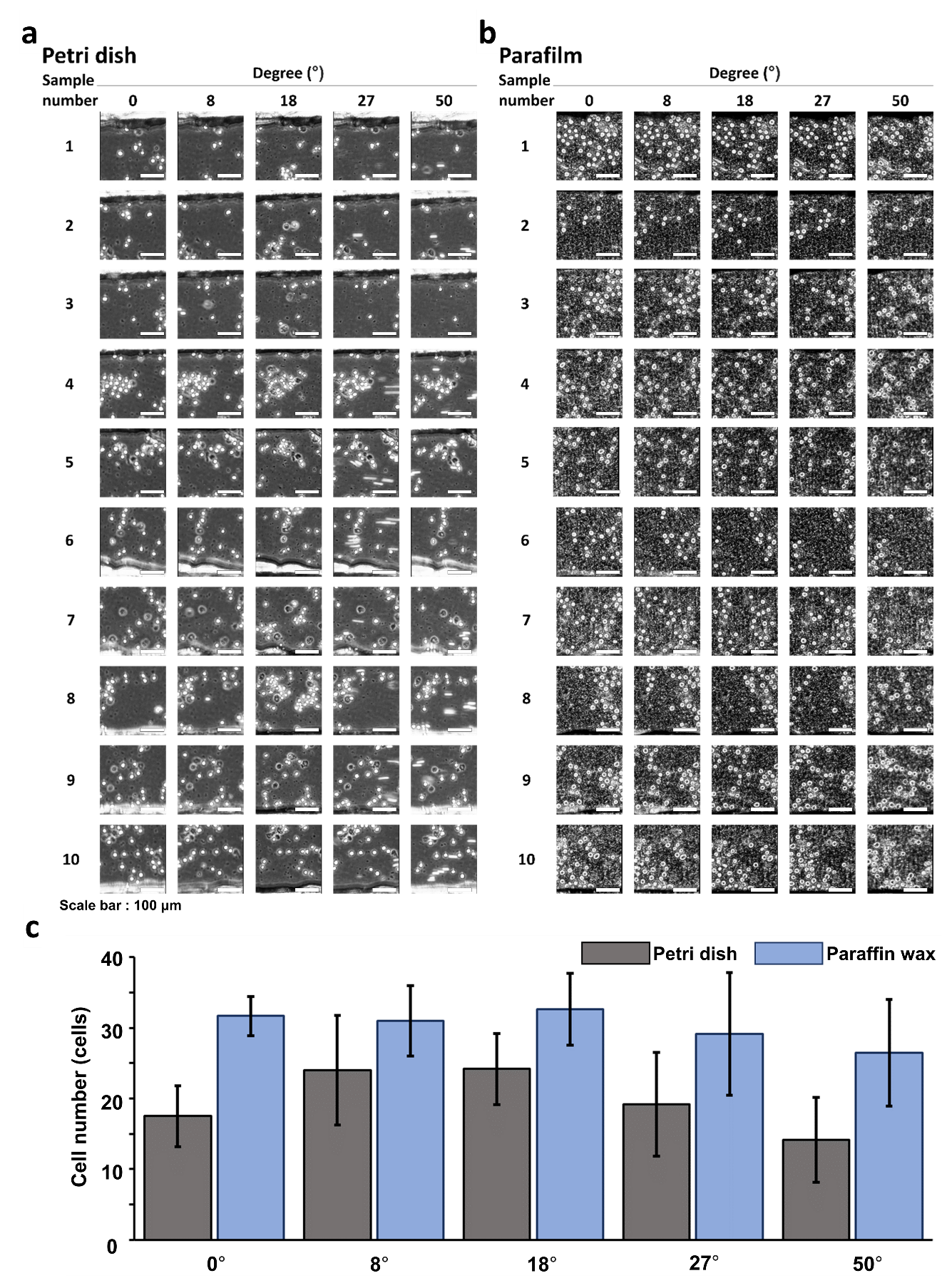


Figure S2. Measurement of changes in cell movement along the cell path with varying angles of repose, as observed using optical microscopy images of the Petri dish (a) and paraffin wax (b). Graph quantifying the number of cells that did not show movement at each angle (c).


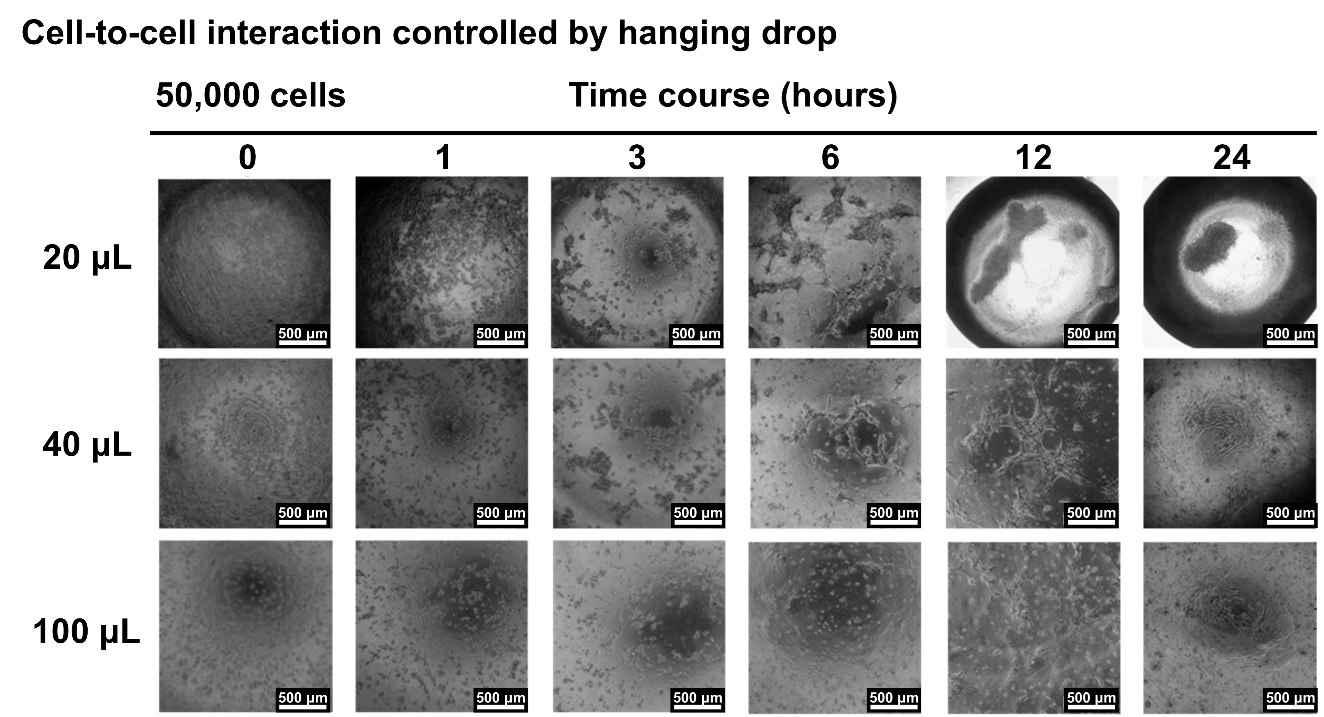


**Figure S3.** Changes in cell formation with increasing cell-to-cell distance using the hanging drop method.


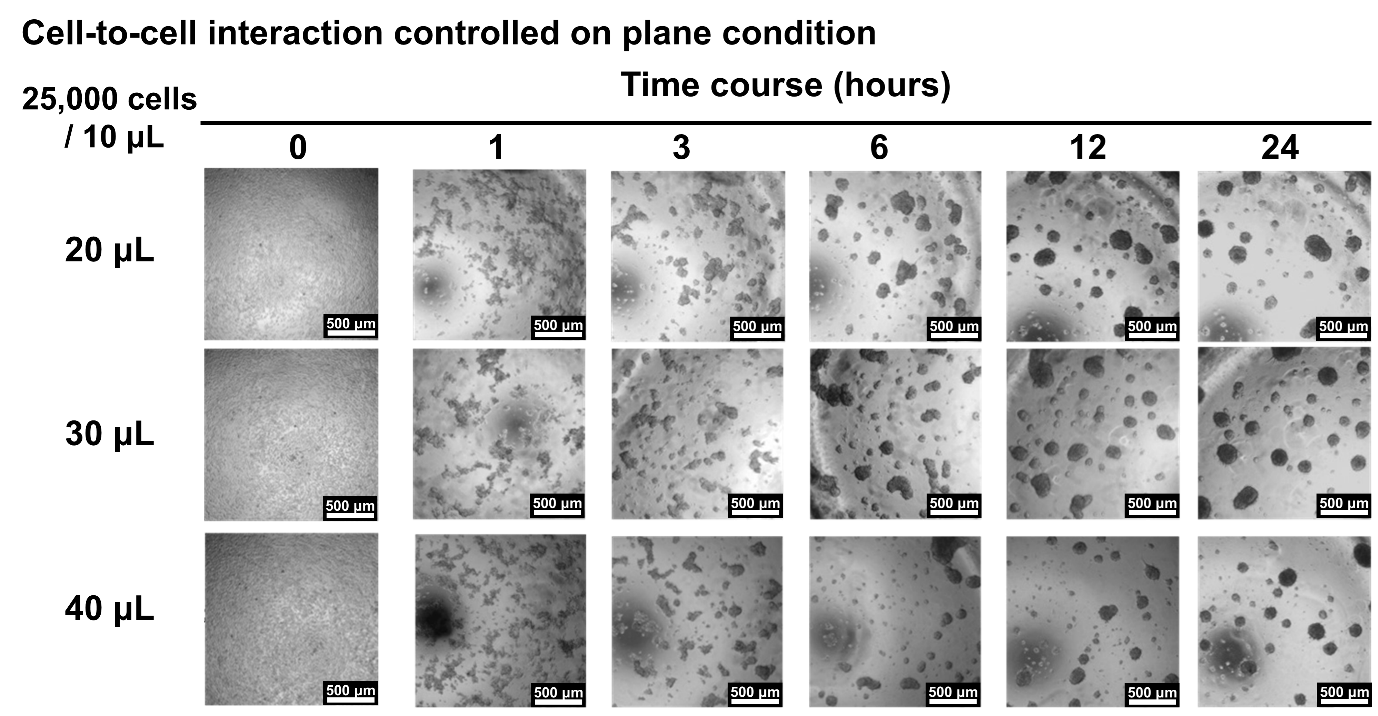


**Figure S4.** Changes in cell formation with increasing cell number, facilitated by maintained cell-to-cell interactions on a planar paraffin wax surface.

**
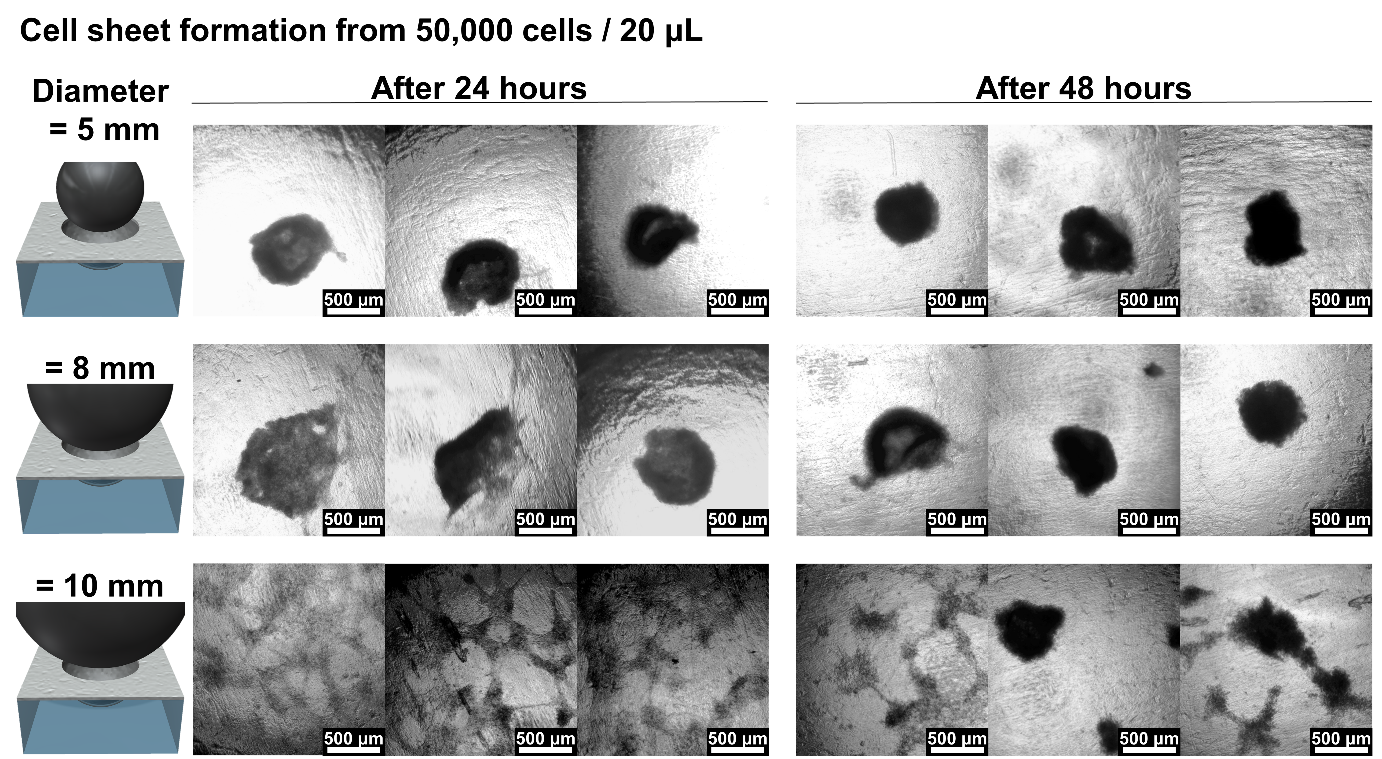
**

Figure S5. A cell sheet was formed using 50,000 cells, according to the curvature of the 3D cell sheet generation, using a hemisphere-like paraffin wax film. The platform was fabricated with stainless steel balls of 5, 8, and 10 mm, while maintaining a hole size of 5 mm to incubate and optically image the cells (three samples for each hole diameter) for 24–48 hours.

**
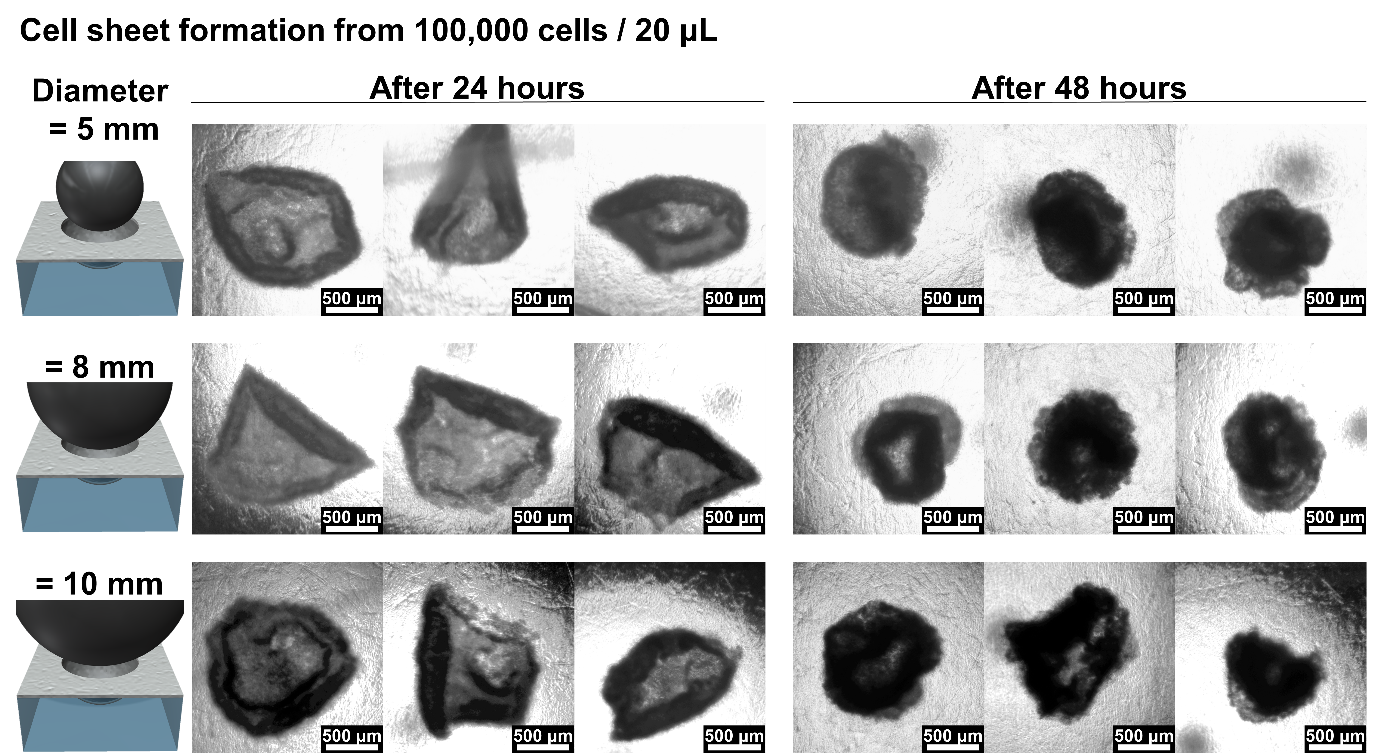
**

Figure S6. A cell sheet was formed using 100,000 cells, according to the curvature of the 3D cell sheet generation, using a hemisphere-like paraffin wax film. The platform was fabricated with stainless steel balls of 5, 8, and 10 mm, while maintaining a hole size of 5 mm to incubate and optically image the cells (three samples for each hole diameter) for 24–48 hours.

**
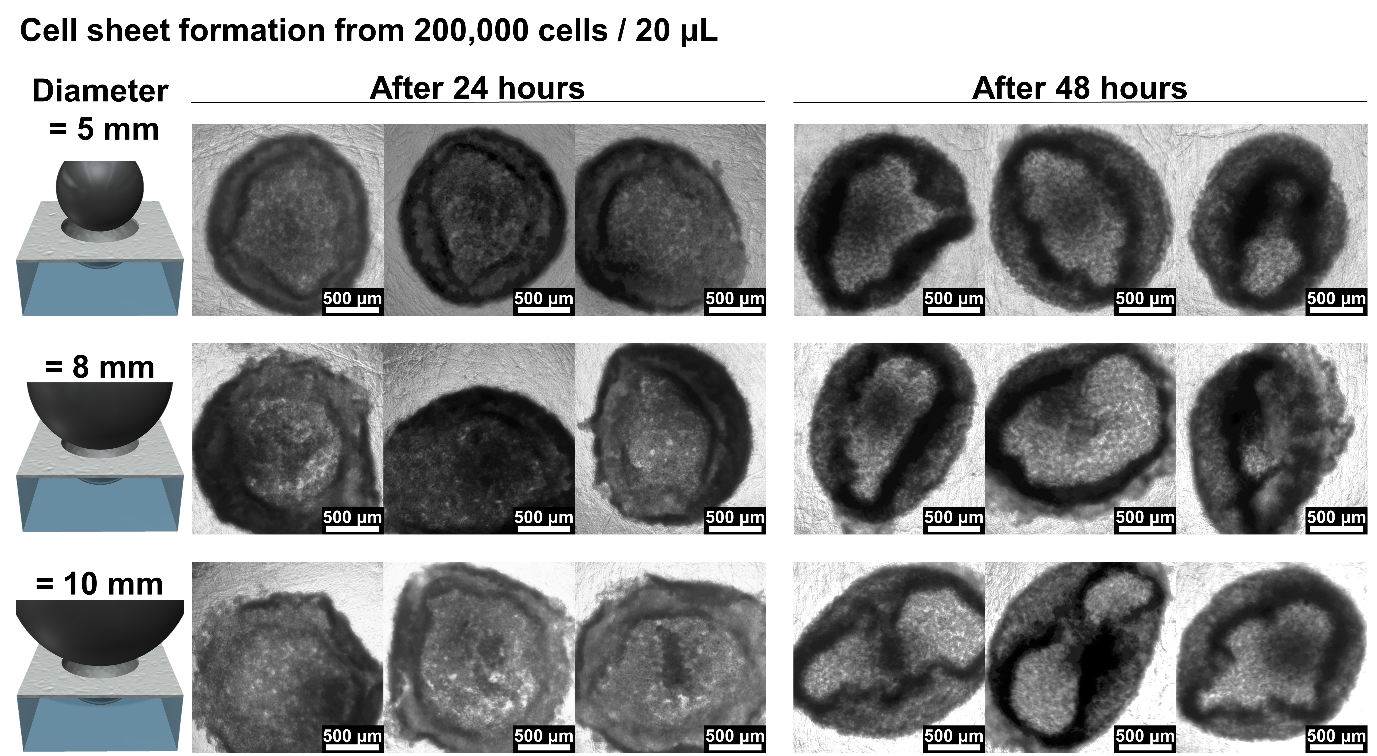
**

Figure S7. A cell sheet was formed using 200,000 cells, according to the curvature of the 3D cell sheet generation, using a hemisphere-like paraffin wax film. The platform was fabricated with stainless steel balls of 5, 8, and 10 mm, while maintaining a hole size of 5 mm to incubate and optically image the cells (three samples for each hole diameter) for 24–48 hours.


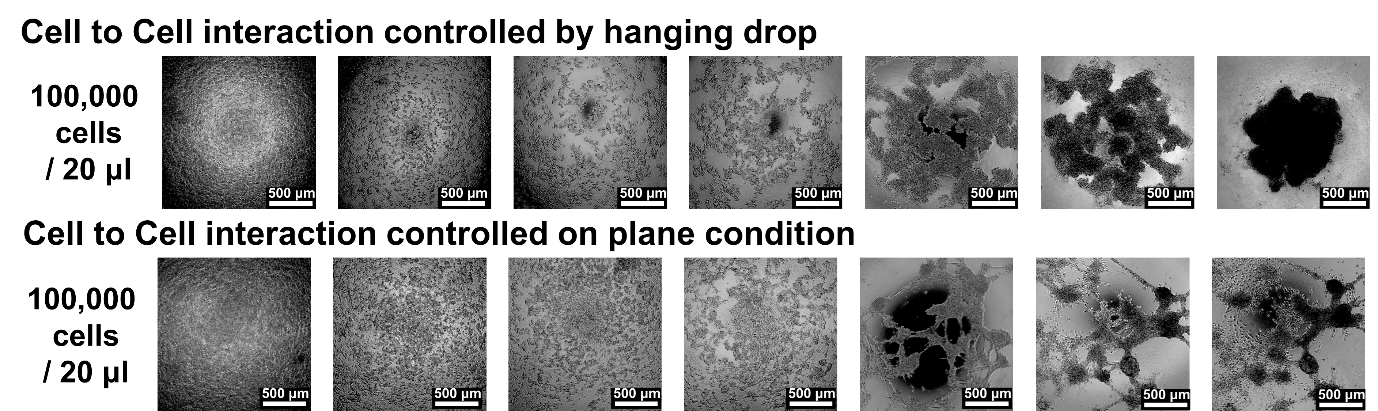


**Figure S8.** Formation of 3D cell structures based on cell-to-cell interactions at a concentration of 100,000 cells per 20 µL.


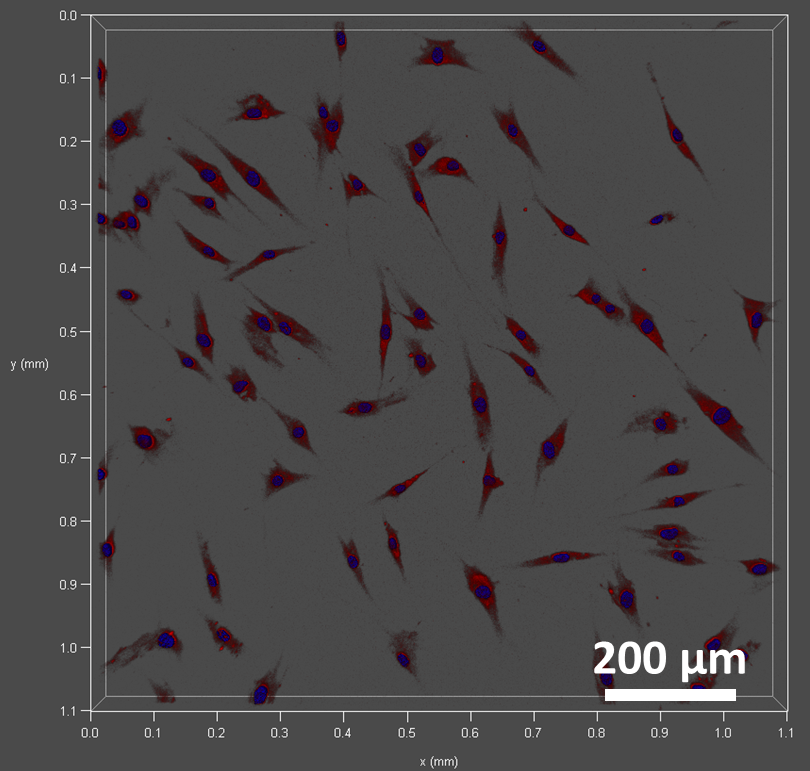


**Figure S9.** The fluorescence image of the 2D cell culture demonstrated the tracking of quantum dot (QD) uptake following cell immobilization, as well as the subsequent cell nuclear staining after QD uptake.
